# Acute Effects of Sugars and Artificial Sweeteners on Small Intestinal Sugar Transport: A Study Using CaCo-2 Cells As an *In Vitro* Model of the Human Enterocyte

**DOI:** 10.1371/journal.pone.0167785

**Published:** 2016-12-16

**Authors:** Patrick O’Brien, Christopher Peter Corpe

**Affiliations:** Diet and Cardiovascular Health Group, Diabetes and Nutritional Sciences Division, King’s College London, London, United Kingdom; Barnard College, UNITED STATES

## Abstract

**Background:**

The gastrointestinal tract is responsible for the assimilation of nutrients and plays a key role in the regulation of nutrient metabolism and energy balance. The molecular mechanisms by which intestinal sugar transport are regulated are controversial. Based on rodent studies, two models currently exist that involve activation of the sweet-taste receptor, T1R2/3: an indirect model, whereby luminal carbohydrates activate T1R2/3 expressed on enteroendocrine cells, resulting in the release of gut peptides which in turn regulate enterocyte sugar transport capacity; and a direct model, whereby T1R2/3 expressed on the enterocyte regulates enterocyte function.

**Aims:**

To study the direct model of intestinal sugar transport using CaCo-2 cells, a well-established *in vitro* model of the human enterocyte.

**Methods:**

Uptake of 10mM ^14^C D-Glucose and D-Fructose into confluent CaCo-2/TC7 cells was assessed following 3hr preincubation with sugars and artificial sweeteners in the presence and absence of the sweet taste receptor inhibitor, lactisole. Expression of the intestinal sugar transporters and sweet-taste receptors were also determined by RT-PCR.

**Results:**

In response to short term changes in extracellular glucose and glucose/fructose concentrations (2.5mM to 75mM) carrier-mediated sugar uptake mediated by SGLT1 and/or the facilitative hexose transporters (GLUT1,2,3 and 5) was increased. Lactisole and artificial sweeteners had no effect on sugar transport regulated by glucose alone; however, lactisole increased glucose transport in cells exposed to glucose/fructose. RT-PCR revealed Tas1r3 and SGLT3 gene expression in CaCo-2/TC7 cells, but not Tas1r2.

**Conclusions:**

In the short term, enterocyte sugar transport activities respond directly to extracellular glucose levels, but not fructose or artificial sweeteners. We found no evidence of a functional heterodimeric sweet taste receptor, T1R2/3 in CaCo-2 cells. However, when glucose/fructose is administered together there is an inhibitory effect on glucose transport possibly mediated by T1R3.

## Introduction

Carbohydrate digestion and absorption occurs primarily in the small intestine (SI). In the SI, sugar oligomers and disaccharides are broken down into monosaccharides by enzymes such as sucrase-isomaltase that are expressed on the apical membrane of the enterocyte. The monosaccharides are then transported into the cell via the Sodium/glucose co-transporter, SGLT1, and the facilitative fructose transporter, GLUT5. Sugars then exit the enterocyte via the facilitative sugar transporter, GLUT2, which is expressed on the basolateral membrane and enter the portal circulation [1]. In order to match assimilation capacity with the luminal carbohydrate load the digestive and transporter mechanisms are tightly regulated: without tight regulation nutrient malabsorption would occur. Chronic regulation (days-weeks) occurs at a developmental, diurnal and pathogenic level, and in general involves control at the gene transcriptional level [2,3]. Acute regulation (<3hrs) also occurs, but the precise mechanisms remain controversial. In rats, when luminal jejunal glucose levels increase, the sweet taste receptor T1R2/3 on the apical membrane of the enterocyte becomes activated, resulting in a rapid and reversible insertion of GLUT2 into the apical membrane which enhances small intestinal sugar transport capacity [4,5]. However, knockout mice studies do not support this acute model: mice null for SGLT1 malabsorb glucose [6] and mice null for GLUT2 have a near normal glucose absorptive capacity [7]. In addition, others have shown T1R2/3 expression to be confined to enteroendocrine cells in the small intestine, indicating regulation of enterocyte transport capacity is indirect, via neighbouring enteroendocrine cells [8].

The detection of the sweet taste receptor, T1R2/3, in tongue taste cells and in small intestine has also resulted in a re-evaluation of the metabolic consequences of artificial sweeteners [9,10]. In rodent studies, artificial sweeteners have been shown to promote small intestinal glucose absorption in the short term [5] as well as glucose intolerance in the long term via alterations in the gut microbiota [11]. *In vitro* studies, using murine enteroendocrine cells (GLUTag cells) have also shown artificial sweeteners promote the release of gut peptide hormones, such GLP-1 and GIP [12] which influence GI function, insulin release and food intake in vivo [13]. In humans, however, evidence of a metabolic effect by artificial sweeteners is contradictory; with some studies indicating no effect on blood glucose excursions and appetite [14] while others have shown they enhance GLP-1 release and lower blood glucose levels [15,16].

The aim of this work was to study the mechanisms that reside in the human enterocyte that are responsible for the acute regulation of intestinal sugar transport capacity by dietary carbohydrates and artificial sweeteners. We hypothesized the sweet-taste receptor, T1R2/3, is expressed in human enterocytes, is functional and is responsible for the acute regulation of sugar transport. To test these hypotheses we used CaCo-2 cells, a well-established *in vitro* model of human enterocytes.

## Materials and Methods

### Cell culture

CacCo-2/TC7 cells were originally obtained from Drs Monique Rousset and Edith Brot-Laroche (CRC, Jussieu, Paris) and have been previously characterised for studies on sugar transport [17,18]. CaCo-2/TC7 cells were grown in 25 mM D-Glucose Dulbecco’s modified Eagle’s medium (DMEM) containing 10% fetal bovine serum, 50 units Penicillin and 50 μg Streptomycin, 0.1 mM MEM non-essential amino acids and additional 2 mM L-Glutamine. Cells were maintained in T25 flasks, split 1:20 once a week at ~80% confluence and seeded for experimentation at a density of 10,000 cells/cm^2^ in 24 multi-well dishes. Medium was changed every other day until confluent and daily after reaching confluence. For experiments cell passages of 38–50 were used.

### Radio-labelled sugar-uptake assays

Assay conditions varied according to the experimental question and any modifications to the following standard protocol are indicated in figure legends/descriptions. Confluent Caco-2/TC7 cells were washed once with Kreb’s buffered saline (KBS, 30 mM HEPES, 130 mM NaCl, 4 mM KH_2_PO_4_, 1 mM MgSO4 and 1 mM CaCl_2_, pH 7.4) and then incubated in KBS containing sugars at 2.5mM, 25 mM or 75 mM and artificial sweeteners at 10 mM in the presence or absence of 0.5 mM lactisole. The incubation medium was then removed and the cells washed once with KBS. Sugar uptake was measured by exposing cells to 10 mM D-Glucose or D-Fructose containing 0.1 μCi/ml [^14^C] radio-labelled sugar as tracer. Cells were lysed with 0.2% SDS for 60 mins at 37°C. Cell lysate were added to scintillation fluid (Ecoscint A) and radioactivity measured by scintillation spectrometry (LS 6500, Beckman-Coulter). Uptake of sugar into cells was determined using the calculated specific activity of the uptake media. Absolute uptake was corrected for simple diffusion by measuring [^14^C] L-Glucose uptake.

### RNA isolation

Confluent Caco-2/TC7 cells were washed with ice-cold PBS and lysed with cold TRIzol^®^ reagent. Chloroform and RNase-free water were added to the cell lysate, rigorously vortexed for 15 s and incubated for 10 mins at room temperature. Samples were then centrifuged at 13,000 RPM at 4°C for 10 mins and the aqueous phase removed and mixed with ice-cold isopropanol and incubated at -20°C for 30 mins. RNA was precipitated by centrifugation at 13,000 RPM at 4°C for 30 min. The resulting pellet was washed twice in 75% ethanol and centrifuged for 5 mins at 13,000 RPM at 4°C. The RNA pellet was resuspended in RNAse-free water, and RNA concentration (ng/μl) and purity (260/280) measured (Nanodrop 2000).

### Reverse Transcription Polymerase Chain Reaction (RT-PCR)

To remove genomic contamination from RNA, 1 μg of RNA sample was incubated with RQ1 RNase-free DNase (Promega, UK) at 37°C for 30 min as indicated by the manufacturer’s protocol. Reverse transcription was performed using a High-Capacity cDNA reverse transcription kit (Applied Biosystems, US) according to the manufacturer’s protocol. The resulting cDNA was then diluted 1:10 for transcriptomic analysis by PCR. Primers were designed using the Roche primer algorithm: slc2a1 5’- ggttgtgccatactcatgacc -3’ with 5’- cagataggacatccagggtagc -3’, predicted amplicon 66 bp, slc2a2 5’- gcatgtgccacactcacac -3’ with 5’- aaaaccagggtcccagtga -3’, amplicon 89 bp, slc2a3 5’- gccctgaaagtcccagattt -3’ with 5’- ttcatctcctggatgtcttgg -3’, amplicon115 bp, slc2a5 5’-tccatttggagggtttatcg -3’ with 5’- aacagcaaggcccctttt -3’, amplicon 78 bp, slc5a1 5’-ctggcaggccgaagtatg -3’ with 5’- ccacttccaatgttactagcaaag -3’, amplicon 68 bp, Slc5a4 5’-aagctgctgcccatgttc -3’ with 5’- cgcattcagaaggtaccacac -3’, amplicon 94 bp, tas1r2 5’-tgaagggcattgttcaccttt -3’ with 5’- gtagcctatcaccttcacttcat -3’, amplicon 91 bp, tas1r3 5’-ttcagtgcaacgcctcag -3’ with 5’- cacgtggaaggtcaggttg -3’, amplicon 89 bp, gnat3 5’-agcgagatgcaagaaccgta -3’ with 5’- cattcttatggatgatcttcatttgt -3’, amplicon 96 bp. All PCRs were run with the FastStart Universal Probe Master mix according to the manufacturer’s protocol using a PTC 200 Peltier Thermal Cycler (MT Research). PCR cycle conditions: 95°C for 15 secs, 40 cycles of 95°C for 15 secs and 60°C for 1 mins, 72°C for 1 min, 4°C forever. PCR products were run on a 2% UltraPure/TBE agarose gel containing Ethidium Bromide run at 120 V for 60 min and visualised on a SynGene Genius Bioimaging system using GeneSnap, version 6.00.26 (Synaptics, Cambridge/UK).

### Statistics

Data analysis and statistics were carried out either in Excel included in Microsoft Office Pro 2007, Version 12.0.6661.5000 SP3 MSO or in GraphPad Prism, Version 6.01. Data are given as arithmetic mean + standard deviation (SD). Significances were tested using the inbuilt two-sided T-Test of Excel, and ANOVA was tested in Prism with a Tukey post-hoc test with Bonferroni correction. A P-value of 0.05 or less was considered statistically significant.

## Results

### D-Glucose and D-Fructose uptake into CaCo-2 cells is via carrier mediated sugar transport

**Fig 1:**
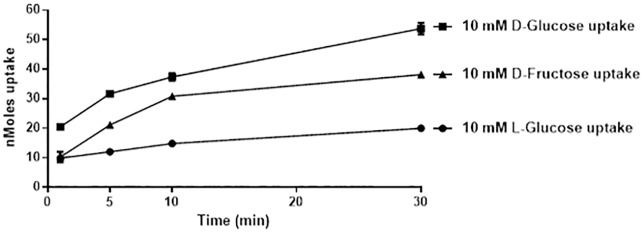
Time course of [14C] 10 mM D-Glucose, D-Fructose and L-Glucose uptake into Caco-2/TC7 cells. Caco-2/TC7 cells grown for 21 days were incubated with 10 mM sugar for 1, 5, 10 or 30 min and cellular uptake measured by radioactive scintillation spectrometry. Data are expressed as nMoles sugar uptake/well ± SD of n = 4 per condition.

The CaCo-2/TC7 sub-clone was used in our studies because it has a higher expression of sugar transporters and hence higher sugar uptake capacity when compared to the parental strain [19]. To validate our *in vitro* system we first wanted to establish a sugar transport assay that was able to distinguish between simple diffusion and carrier mediated sugar transport. To do this, confluent CaCo-2 cells were incubated in 10 mM ^14^C D-Glucose, D-Fructose or L-Glucose and accumulation of radioactivity inside cells measured as a function of the time of incubation (1–30 min). L-Glucose is not transported into cells by carrier mediated transport but by simple diffusion and its uptake was linear between 1 and 30 mins (Fig 1). D-Glucose and D-Fructose enters the cell via carrier mediated and simple diffusion and uptake was readily detected above simple diffusion. Subtracting total D-glucose and D-Fructose uptake from L-Glucose uptake gives the carrier mediated component of sugar transport. For all sugar transport assays presented we measured sugar uptake at 10 mins and subtracted the simple diffusion component (L-Glucose uptake) to give a measure of carrier mediated transport.

### Transcripts for the Na^+^ dependent glucose transporter, SGLT1, and the facilitative sugar transporters, GLUT1, 2, 3 and 5 were detected in CaCo-2/TC7 cells

**Fig 2:**
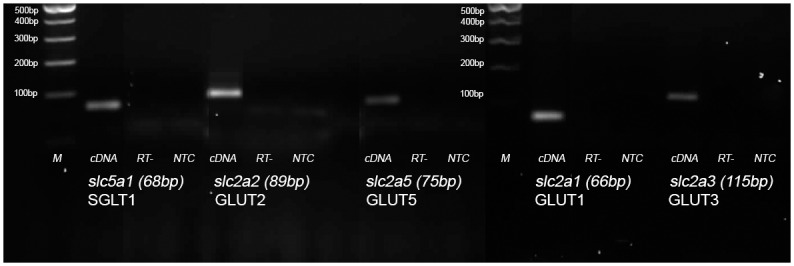
Identification of hexose transporter genes expressed in Caco-2/TC7 cells. RNA was isolated from Caco-2/TC7 cells grown for 21 days and converted to cDNA. The presence of SGLT1 (slc5a1), GLUT2 (slc2a2), GLUT5 (slc2a5), GLUT1 (slc2a1) and GLUT3 (slc2A3) transcripts was undertaken by PCR. (M) represents the molecular weight marker ladder. For each gene, (cDNA) represents a PCR amplification using Caco-2/TC7 cDNA as a template, (RT-) represents a PCR using template obtained from reverse transcriptase reaction without enzyme. (NTC) represents a PCR using no template.

To identify which hexose transporters are present and contributing to the observed carrier mediated sugar transport RT-PCR was performed on differentiated CaCo-2/TC7 cells. Amplicons of the correct predicted nucleotide size were obtained for human slc5a1, slc2a1, slc2a2, slc2a3 and slc2a5 (Fig 2). No amplicons were detected by PCR using RT- as template or in the no-template control, indicating PCRs were clean and devoid of potential genomic contamination. Based on previous reports [18] and the presented PCRs, human SGLT1, GLUT1, GLUT2, GLUT3 and GLUT5 are expressed in Caco-2/TC7 cells and contributing towards the detected carrier mediated transport of sugars.

### In response to changing concentrations of D-Glucose, but not D-Fructose, in the extracellular medium CaCo-2/TC7 cells acutely regulate glucose and fructose transport capacity

**Fig 3:**
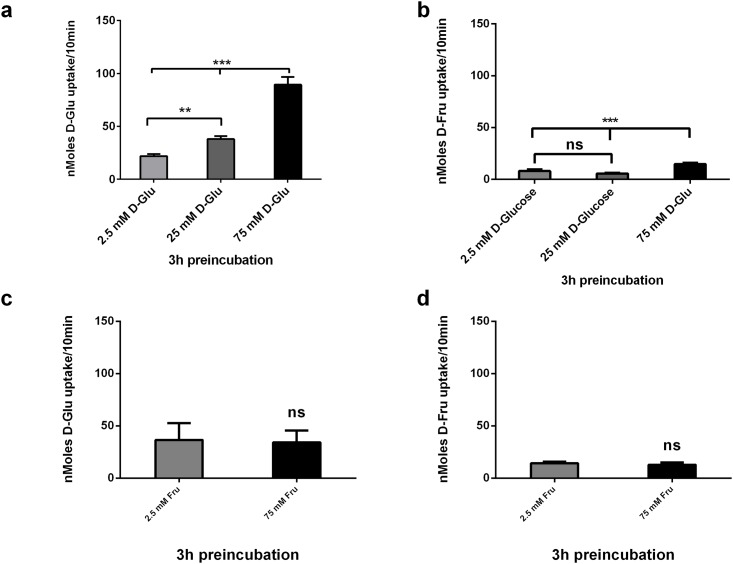
Acute effects of D-Glucose and D-Fructose on carrier mediated [^14^ C] 10 mM D-Glucose and D-Fructose uptake into Caco-2/TC7 cells. Caco-2/TC7 cells grown for 21 days were incubated with 2.5, 25 and 75 mM D-Glucose or D-Fructose for 3hrs, washed in sugar free KBS, followed by 10 mins exposure to (a and c) [^14^ C] 10 mM D-Glucose and (b and d) [^14^C] 10 mM D-Fructose. Cellular uptake of D-Glucose and D-Fructose was measured by radioactive scintillation spectrometry. Osmolarity was adjusted by adding Mannitol. Uptake is corrected for simple diffusion of 10 mM [^14^C] L-Glucose. Data are expressed as nMoles D-Glucose or D-Fructose uptake/well/10min ± SD of n = 4 per condition. *P < 0.05, **P < 0.01, ***P < 0.001, ns = not significant.

To establish if the sugar transport mechanisms in CaCo-2 cells respond in the short term to changes in substrate concentrations in the external media, differentiated CaCo-2/TC7 cells were incubated with 2.5, 25 mM and 75 mM D-Glucose or D-Fructose in KBS for 3hrs and then [^14^C] 10 mM D-Glucose and D-Fructose uptake was measured. CaCo-2/TC7 cells were maintained in 25 mM D-Glucose DMEM which is fructose free. Switching from 25 mM to 2.5 mM D-Glucose in KBS for 3hrs resulted in a significant 60% decrease in 10 mM D-Glucose uptake, but no change in D-Fructose uptake (Fig 3a and 3b); whereas switching from 25 mM to 75 mM D-Glucose resulted in a significant 3-fold increase in D-Glucose and D-Fructose uptake (Fig 3a and 3b). In contrast, switching the incubation media from 25 mM D-Glucose (DMEM) to D-Fructose (2.5mM and 75 mM) had no impact on D-Glucose or D-Fructose transport capacity (Fig 3c and 3d). These data indicate that under the conditions employed sugar transport by CaCo-2 cells acutely respond to changes in extracellular glucose, but not fructose.

### The T1R3 inhibitor, lactisole, and artificial sweeteners have no effect on the acute regulation of glucose transport in CaCo-2 cells

**Fig 4:**
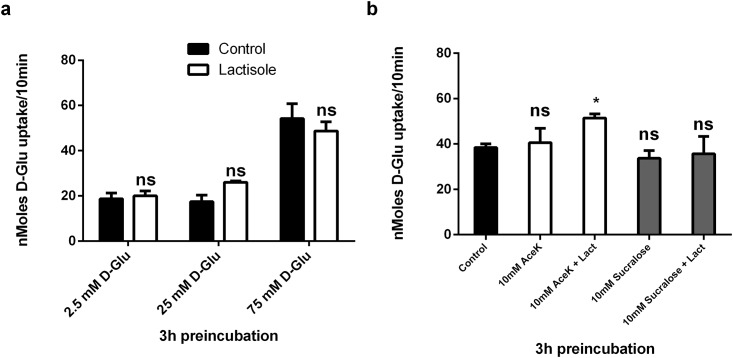
Acute effects of the sweet taste inhibitor, lactisole and artificial sweeteners on carrier mediated glucose transport in Caco-2/TC7 cells. (a) Caco-2/TC7 cells grown for 21 days were incubated with 2.5, 25 and 75 mM D-Glucose for 3hrs in the presence or absence of 0.5 mM lactisole, washed three times with glucose free KBS, followed by 10 min exposure to [^14^ C] 10 mM D-Glucose. Osmolarity was adjusted by adding Mannitol. (b) Cells were incubated with 75 mM D-Glucose in the presence of 10 mM AcesulfameK (AceK) or Sucralose with the addition of lactisole at 0.5mM. Osmolarity was adjusted for by adding Mannitol. Cellular uptake of D-Glucose was measured by radioactive scintillation spectrometry. Uptake is corrected for simple diffusion of 10 mM [^14^C] L-Glucose. Data are expressed as nMoles D-Glucose uptake/well/10min ± SD of n = 4 per condition. ns = not significant.

To determine if the sweet-taste receptor, T1R2/3, was responsible for the acute upregulation of sugar transport by D-Glucose, we measured 10 mM D-Glucose uptake in CaCo-2/TC7 cells preincubated for 3hrs with 2.5 mM, 25 mM and 75 mM D-Glucose in the presence and absence of 0.5 mM of the T1R3 inhibitor, lactisole [20]. The glucose mediated upregulation in glucose transport activity was not inhibited by lactisole (Fig 4a) indicating T1R3 (and thus, the heterodimeric, sweet taste receptor, T1R2/3) is not responsible for acute regulation of sugar transport in CaCo-2 cells. Lactisole also had no effect on fructose transport (S1 Fig). To further test the presence of a functional sweet taste receptor in our system we studied the acute effects of sweet taste receptor agonists in the form of the artificial sweeteners on D-Glucose transport. Caco2 cells were preincubated for 3hrs with 10 mM AceK and Sucralose in the presence/absence of lactisole and then 10 mM ^14^C D-Glucose transport activity measured. D-Glucose uptake was not enhanced by the added artificial sweeteners, nor was transport inhibited by lactisole (Fig 4b). These data suggest artificial sweeteners do not influence intestinal sugar transport by direct regulation of the sugar transporters expressed on enterocytes.

### Tas1r3 transcripts are expressed in CaCo-2 cells, but not Tas1r2 or Gnat3 transcripts

**Fig 5:**
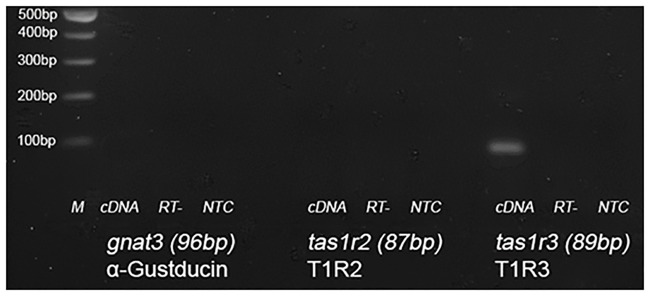
Identification of sweet taste receptor transcripts expressed in Caco-2/TC7 cells. RNA was isolated from Caco-2/TC7 cells grown for 21 days and converted to cDNA. The presence of α-Gustducin (gnat3), T1R2 (tas1r2) and T1R3 (tas1r3) transcripts was undertaken by PCR. (M) represents the molecular weight marker ladder. For each gene, (cDNA) represents a PCR amplification using Caco-2/TC7 cDNA as a template, (RT-) represent a PCR using template obtained from a reverse transcriptase reaction without enzyme. (NTC) represents a PCR using no template.

The heterodimeric sweet taste receptor, T1R2/3 has been detected in rodent small intestine. However, its precise cellular localization remains controversial, with some groups detecting it in human and rodent enteroendocrine cells in duodenum [8] and others in rodent jejunal enterocytes [5]. To determine if the sweet taste receptor T1R2/3 is expressed in human enterocytes, we assessed the presence of Tas1r2 and 3 and Gnat3 transcripts by RT-PCR in CaCo-2/TC7 cells. An amplicon of the correct predicted nucleotide size was detected for Tas1r3; however, no amplicon was detected for Tas1r2 or the downstream signalling molecule, Gnat3 (Fig 5). RT- and no template controls did not yield amplicons indicating our PCR was clean and free from genomic contamination. RT-PCR using human tongue cDNA as template yielded amplicons for Tas1r2 and r3, indicating primers and PCR conditions were appropriate (S2 Fig). These data suggest CaCo-2/TC7 cells (and possibly human enterocytes) do not express a functional heterodimeric sweet taste receptor, T1R2/3.

### An equimolar mix of free glucose and fructose acutely regulates glucose transport activity and it is potentiated by lactisole in CaCo-2 cells

**Fig 6:**
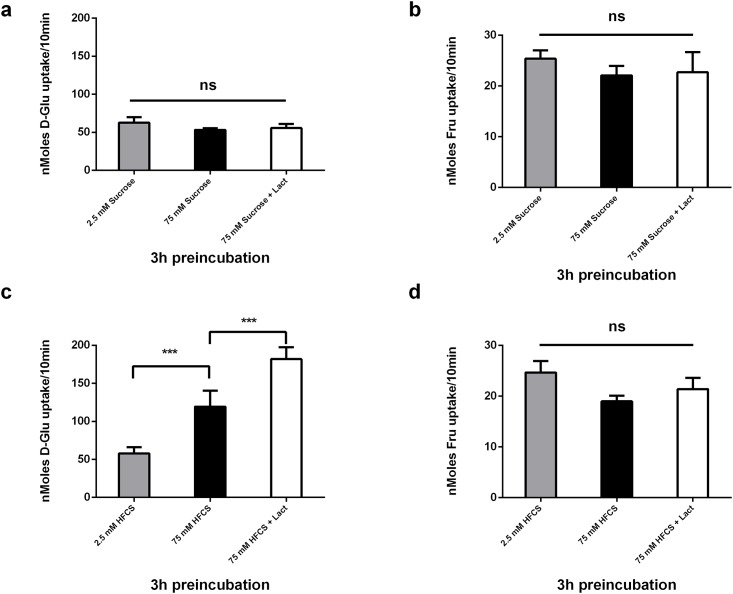
Acute effects of Sucrose, an equimolar mix of free glucose and fructose, and lactisole on carrier mediated [^14^C] 10 mM D-Glucose and D-Fructose uptake into Caco-2/TC7 cells. Caco-2/TC7 cells grown for 21 days were incubated with 2.5 and 75 mM Sucrose or a 50/50 mix of free D- Glucose/D-Fructose in the presence or absence of 0.5 mM lactisole for 3hrs, washed in sugar free KBS. (a) and (c) are cellular uptake of [^14^C] 10 mM D-Glucose for 10 mins (b) and (d) are cellular uptake of [^14^C] 10 mM D-Fructose measured by radioactive scintillation spectrometry. Osmolarity was adjusted by adding Mannitol. Uptake is corrected for simple diffusion of 10 mM [^14^C] L-Glucose. Data are expressed as nMoles sugar uptake/well/10min ± SD of n = 4 per condition. *P < 0.05, **P < 0.01, ***P < 0.001, ns = not significant.

A previous report using HEK293 cells transfected with Tas1r3 suggested T1R3 acted as a functional sucrose sensor [21]. To test whether the Tas1r3 gene expression in CaCo-2/TC7 cells resulted in a functional T1R3 sucrose sensor we preincubated the cells for 3 hrs with 2.5 and 75 mM sucrose in the presence or absence of lactisole and measured 10 mM D-Glucose and D-Fructose transport. Sugar transport was not stimulated by the presence of sucrose in the pre-incubation medium, and there was no effect of the lactisole (Fig 6a and 6b). Preincubation with an equimolar mix of free D-Glucose and D-Fructose did however stimulate glucose transport; furthermore, the sweet taste inhibitor, lactisole, potentiated the effect (Fig 6c). The equimolar mix of glucose/fructose did not have an effect on D-Fructose nor did lactisole (Fig 6d).

## Discussion

The molecular mechanisms responsible for the regulation of small intestinal sugar transport remain unclear. From rodent studies, two models currently exist that involve activation of the sweet-taste receptor, T1R2/3. Margolskee’s group showed T1R2/3 to be expressed exclusively in murine small intestinal enteroendocrine cells, and an important regulator of the intestinal response to chronic changes in dietary carbohydrate levels: following 2 wks on a high carbohydrate diet an increase in SGLT1 mediated glucose transport capacity was detected in wild type mice, but not in mice null for the Tas1r3 or alpha gustducin genes [8,12]. In contrast, Kellett’s group detected T1R2/3 expression in rat jejunal enterocytes, and short term exposures of sugars and artificial sweeteners promoted intestinal glucose transport capacity via T1R2/3 dependent insertion of GLUT2 into the apical membrane of enterocytes [5]. At one level the two models are not mutually exclusive: chronic changes in small intestinal glucose absorptive capacity are mediated by changes in SGLT1 gene and protein expression levels in enterocytes; whereas acute changes are mediated by the control of GLUT2 protein expression levels on the apical membrane of the enterocyte. The differences in the cellular localization of T1R2/3 are however difficult to reconcile: the Margolskee model suggests regulation of small intestinal sugar transport capacity is indirect, via T1R2/3 expressed on enteroendocrine cells regulating neighbouring enterocytes; whereas the Kellett model suggest regulation is direct, via T1R2/3 expressed on the enterocytes apical membrane.

To study the short term regulation of intestinal sugar transport capacity by dietary carbohydrates and artificial sweeteners and to establish the role the sweet-taste receptor T1R2/3 plays in direct regulation of sugar transport, we used the CaCo-2/TC7 cell line, a well-established *in vitro* model of human enterocytes. Carrier mediated transport of D-glucose and D-fructose was readily detected that was mediated by a combination of the transport activities of SGLT1, GLUT1, 2, 3 and 5. Furthermore, our data suggest when extracellular glucose levels are increased from 2.5 mM to 75 mM the transport activities of SGLT1 and/or the facilitative glucose transporters (GLUT1, GLUT2 and/or GLUT3) are increased; and when increased from 25 mM to 75 mM transport activity of the glucose/fructose transporter GLUT2 is increased. Changes in the transport activity of the fructose transporter, GLUT5, could also explain the increase in fructose transport, although we think this is unlikely because dietary glucose does not regulate fructose transport capacity via GLUT5, at least in rodents [22]. In support of our *in vitro* findings, glucose transport (km and Vmax) is decreased when CaCo-2 cells are starved of glucose for 1 hr, and GLUT2 trafficking to the apical membrane is increased when extracellular levels of glucose are increased to 50 mM [23]. Collectively, these *in vitro* data suggest human enterocytes are able to respond directly to extracellular glucose, which is consistent with the model of acute regulation of sugar transport proposed by the Kellett group.

The upregulation in fructose transport activity by glucose may have clinical relevance. In humans, fructose absorption is much slower than for glucose [24]. In addition it has been estimated that more than half of the population display symptoms of malabsorption when dietary fructose levels are between 25-50g [25]. Although, the precise mechanisms responsible for fructose malabsorption are unknown, it can be treated by the addition of glucose [25]. The CaCo-2 cell data presented in this report suggest dietary glucose may promote fructose clearance from the gut by upregulating fructose transport activity via an increase in the expression of the glucose/fructose transporter, GLUT2 (or possibly GLUT5) in the apical membrane.

When we pre-incubated CaCo-2 cells with 2.5 mM and 75 mM fructose for 3hrs no change in fructose (or glucose) transport activity was detected. The absence of a fructose effect is inconsistent with the *in vitro* effects of glucose reported herein and also from rodent studies, which have shown acute (3hrs) and chronic (1–3 weeks) exposure to diets high in fructose result in an upregulation in small intestinal fructose transport capacity mediated by an increase in GLUT5 mRNA and protein expression levels [26,27]. The reason for the discrepancy is unclear. It is possible there is a species difference in the response to dietary fructose, and CaCo-2 cells simply reflect an inability of the human small intestine to adapt to changes in dietary fructose levels, at least in the short term. It may also be that regulation of intestinal fructose transport by dietary levels of fructose requires signalling between neighbouring enteroendocrine cells and enterocytes, which is clearly absent in our CaCo-2 system.

In this study the sweet taste receptor inhibitor, lactisole, did not inhibit sugar transport. We also analysed the expression of Tas1r2, Tas1r3 and Gnat3 transcripts in Caco-2/TC7 cells, but were only able to detect Tas1r3 transcripts. Our data suggest CaCo-2 cells (and thus human enterocytes) do not possess a functional heterodimeric sweet taste receptor, T1R2/3, which is in contrast to the Kellett model. Using the same cell line, Le Gall et al [28] showed lactisole blocked the upregulation of SGLT1 and GLUT5 mRNA expression levels following chronic (2 days) exposure to dietary carbohydrates. Their experimental conditions were however very different to ours: they starved differentiated CaCo-2/TC7 cells for 2 days prior to performing experiments. Not only is this unphysiological, but from our experience it is detrimental to cell health/survival; 48 hr starvation resulted in mono-layer detachment and cell death. They also detected Tas1r3 and Gnat3 by RT-PCR (Tas1r2 gene expression was not studied). In addition, immunohistochemical analysis showed T1R2 and T1R3 proteins present on CaCo-2 cell membranes. It is possible that differences in passage number may have resulted in the loss of Tas1r2 and Gnat3 gene expression in our cells.

The effects of artificial sweeteners on intestinal sugar transport are controversial, with conflicting reports on their effects obtained from *in vitro*, rodent and human studies [5,10,12,14–16]. Artificial sweeteners had no effect on sugar transport in CaCo-2/TC7 cells, which is consistent with data collected using the parental CaCo-2 cell strain [23]. Interestingly, in the same report the authors claim to have detected a response by artificial sweeteners at higher glucose concentrations (>25 mM) although no fold change data were given and no statistical analysis was performed to support their conclusions. Although artificial sweeteners had no direct effect on sugar transport in the short term, there may be a long term effect on enterocyte transport capacity via changes in transporter gene expression levels [28] or an indirect effect via interactions with the sweet taste receptor in enteroendocrine cells.

In short term (60 s) drinking studies, Tas1r3 knockout mice have diminished preference for sucrose solutions (0–32%) when compared to wild type controls, indicating the importance of T1R3 in the taste-mediated response to sucrose [29]. A previous report using HEK293 cells transfected with Tas1r3 showed T1R3 alone can act as a functional sucrose sensor, albeit at very high concentrations of sucrose (500mM)[21]. Since Tas1r3 transcripts were detected by PCR in CaCo-2/TC7 cells, T1R3 protein might be expressed on the plasma membrane (indeed this has been shown by others (28)) and functioning as a low affinity sucrose sensor. Incubating CaCo-2 cells for 3hrs with increasing concentrations of sucrose (2.5 mM and 75 mM) did not however elicit a change in glucose or fructose transport activity, nor was there a response to 0.5 mM lactisole treatment. It is possible a T1R3 mediated response could have been detected at sucrose concentrations of >300 mM, although this would have been unphysiologic. The absence of a sucrose effect might also be because acute upregulation of sugar transport by human enterocytes requires free glucose to reach a threshold of > 25–30 mM [30]. Sucrose has to be broken down by sucrase-isomaltase, which could be rate limiting in the delivery of free glucose (and fructose) to the plasma membrane. Indeed, when rodents are chronically fed diets high in glucose and fructose, small intestinal SGLT1 and GLUT5 gene and protein expression levels are increased, respectively, but diets high in sucrose have no effect on transporter expression levels [22]. If correct, acute trafficking of GLUT2 to the apical membrane may not occur in foods containing complex carbohydrates due to the rate limiting activities of amylase and the dissacharidases.

In this report we have shown that changes in extracellular glucose levels (but not fructose levels) result in an upregulation in glucose and fructose transport activity that is independent of T1R2/3 activation. However, humans rarely ingest glucose or fructose alone: free glucose and fructose will be present in a variety of proportions in fruits and in HFCS, and are covalently linked together in sucrose. We therefore assessed the effects of a mixture of free glucose and fructose on sugar sensing and transport. Following exposure to a 50/50 mix of fructose/glucose, glucose transport but not fructose transport was increased. These data are in contrast to the data collected when cells were exposed to the individual sugars and suggests the sugar mix increases glucose transport activities of SGLT1 and GLUT1/GLUT3 alone, but inhibits the upregulation in GLUT2 and or GLUT5. Surprisingly, lactisole enhanced glucose but not fructose transport, suggesting fructose may be dampening increased glucose transport activities via T1R3 activation. If correct, this intriguing result provides an alternative mechanistic explanation for fructose malabsorption: luminal fructose has an inhibitory effect on intestinal glucose transporters (principally SGLT1) via T1R3 signalling leading to glucose malabsorption. Addition of glucose or changing the ratio of ingested fructose/glucose in favour of glucose overcomes the inhibition by dietary fructose.

Another sugar sensing mechanism may be responsible for the detected responses to glucose. SGLT3 has a high specificity for D-Glucose but does not transport the sugar across the plasma membrane, instead glucose binding causes Na+ influx and membrane depolarization, and has thus been described as a putative intestinal sugar. SGLT3 was first detected in intestinal cholinergic neurons and smooth muscle [31]. We have detected SGLT3 gene expression in CacCo-2 cells by RT-PCR (data not shown). More research is needed to determine its precise role in enterocyte as well as intestinal function.

In summary, our *in vitro* data show an acute regulation of glucose and fructose transport by extracellular glucose, independent to T1R2/3 activation in enterocytes. We also show that artificial sweeteners do not directly influence glucose or fructose transport activities. Although our short term studies did not detect a functional heterodimeric sweet taste receptor, T1R2/3 in CaCo-2 cells, T1R3 and SGLT3 may be functional.

## Supporting Information

S1 Fig Acute effects of lactisole on carrier mediated fructose transport in Caco-2/TC7 cells. (a) Caco-2/TC7 cells grown for 21 days were incubated with 75 mM D-Fructose for 3hrs in the presence or absence of 0.5 mM lactisole, washed three times with glucose free KBS, followed by 10 min exposure to [^14^ C] 10 mM D-Fructose. Osmolarity was adjusted by adding Mannitol. Cellular uptake of D-Fructose was measured by radioactive scintillation spectrometry. Uptake is corrected for simple diffusion of 10 mM [^14^C] L-Glucose. Data are expressed as nMoles D-Fructose uptake/well/10min ± SD of n = 4 per condition. ns = not significant.
(TIF)

S2 Fig Identification of sweet taste receptor genes expressed in human tongue. The presence of ß-actin, T1R2 (tas1r2) and T1R3 (tas1r3) transcripts in human tongue cDNA (Clontech) was assessed by PCR. (M) represents the molecular weight marker ladder. For each gene, (cDNA) represents a PCR amplification using tongue cDNA as a template, (RT-) represent a PCR using template obtained from a reverse transcriptase reaction without enzyme. (NTC) represents a PCR using no template.
(TIF)
