# Correction: Acute Effects of Sugars and Artificial Sweeteners on Small Intestinal Sugar Transport: A Study Using CaCo-2 Cells As an *In Vitro* Model of the Human Enterocyte

**DOI:** 10.1371/journal.pone.0186016

**Published:** 2017-10-03

**Authors:** Patrick O’Brien, Christopher Peter Corpe

Incorrect versions of Figs 3, 4 and 6 are included in the published paper.

In Fig 3B and 3D, the Y-axis scale should be adjusted for clarity. Please see the corrected Fig 3 here.

**Fig 3:**
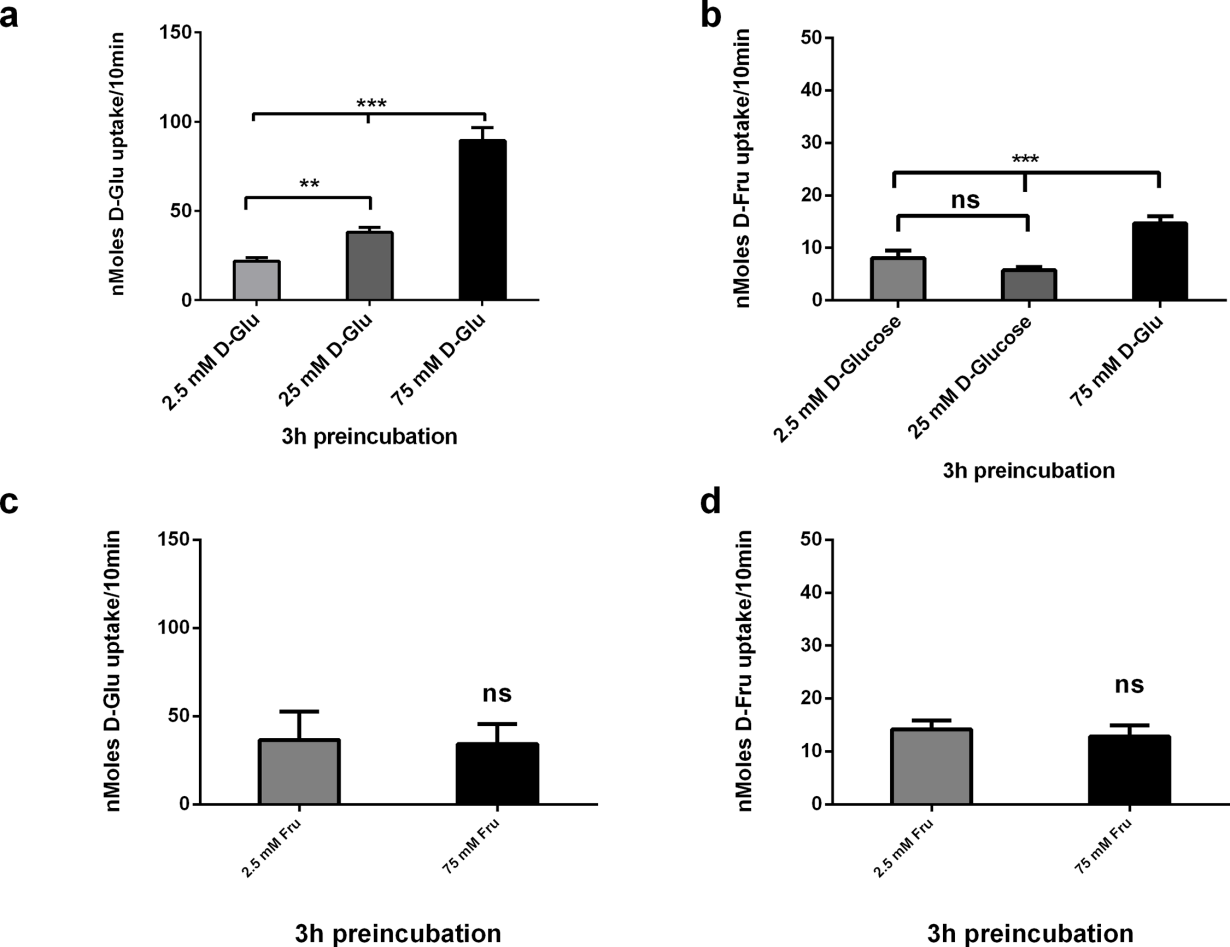
Acute effects of D-Glucose and D-Fructose on carrier mediated [^14^ C] 10 mM D-Glucose and D-Fructose uptake into Caco-2/TC7 cells. Caco-2/TC7 cells grown for 21 days were incubated with 2.5, 25 and 75 mM D-Glucose or D-Fructose for 3hrs, washed in sugar free KBS, followed by 10 mins exposure to (a and c) [^14^ C] 10 mM D-Glucose and (b and d) [^14^C] 10 mM D-Fructose. Cellular uptake of D-Glucose and D-Fructose was measured by radioactive scintillation spectrometry. Osmolarity was adjusted by adding Mannitol. Uptake is corrected for simple diffusion of 10 mM [^14^C] L-Glucose. Data are expressed as nMoles D-Glucose or D-Fructose uptake/well/10min ± SD of n = 4 per condition. *P < 0.05, **P < 0.01, ***P < 0.001, ns = not significant.

In Fig 4B, 10 mM AceK+Lact is not significant when compared to 10 mM AceK alone. In the published Fig 4B, the significance (*) is an incorrect comparison between 10 mM AceK+Lact and the control. Please see the corrected Fig 4 here.

**Fig 4:**
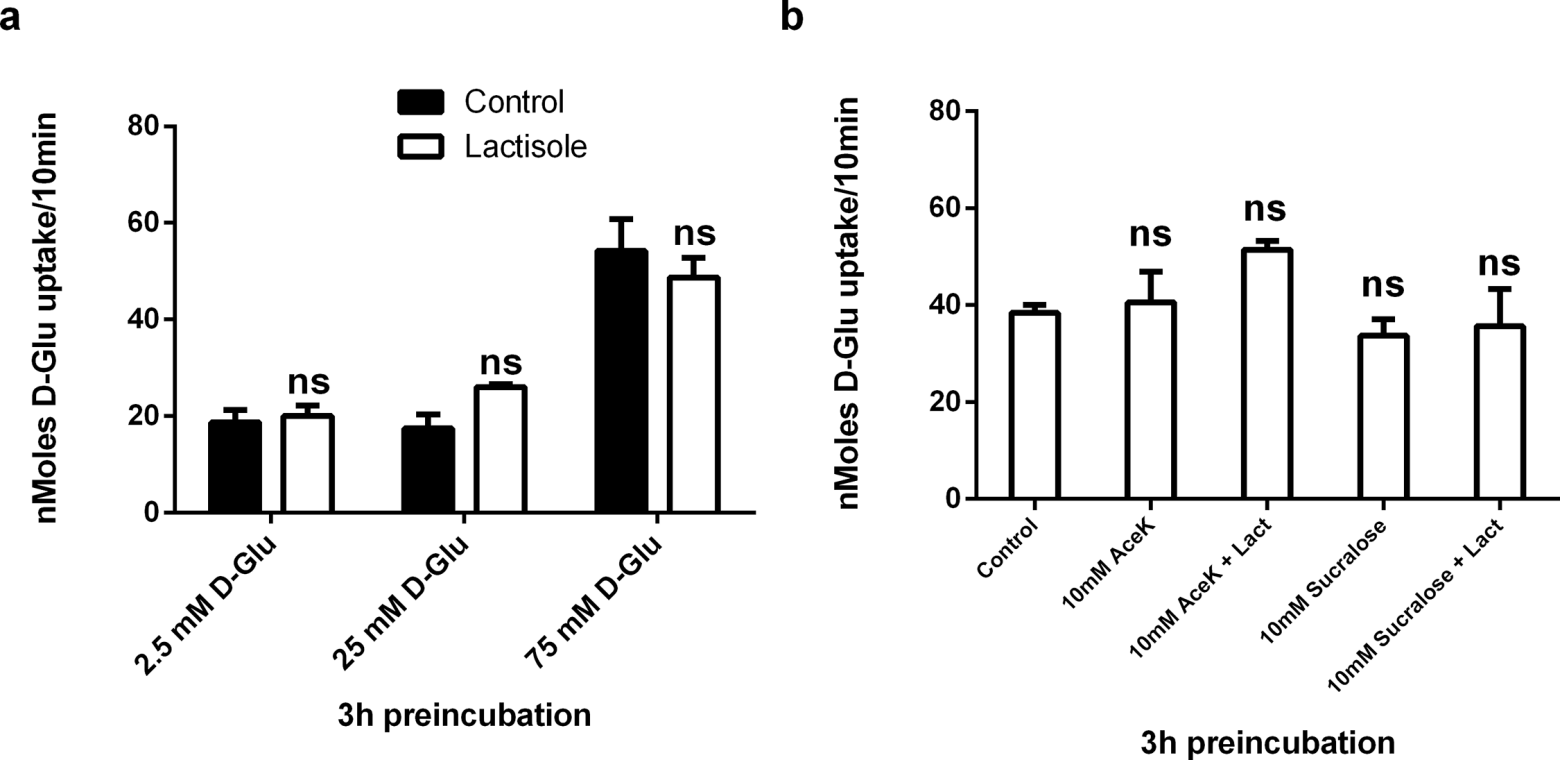
Acute effects of the sweet taste inhibitor, lactisole and artificial sweeteners on carrier mediated glucose transport in Caco-2/TC7 cells. (a) Caco-2/TC7 cells grown for 21 days were incubated with 2.5, 25 and 75 mM D-Glucose for 3hrs in the presence or absence of 0.5 mM lactisole, washed three times with glucose free KBS, followed by 10 min exposure to [^14^ C] 10 mM D-Glucose. Osmolarity was adjusted by adding Mannitol. (b) Cells were incubated with 75 mM D-Glucose in the presence of 10 mM AcesulfameK (AceK) or Sucralose with the addition of lactisole at 0.5mM. Osmolarity was adjusted for by adding Mannitol. Cellular uptake of D-Glucose was measured by radioactive scintillation spectrometry. Uptake is corrected for simple diffusion of 10 mM [^14^C] L-Glucose. Data are expressed as nMoles D-Glucose uptake/well/10min ± SD of n = 4 per condition. ns = not significant.

In Fig 6C and 6D, the x-axis is labelled as HFCS but should be labeled as D-Glu + D-Fru. The y-axis scale should also be adjusted for clarity. Please see the corrected Fig 6 here.

**Fig 6:**
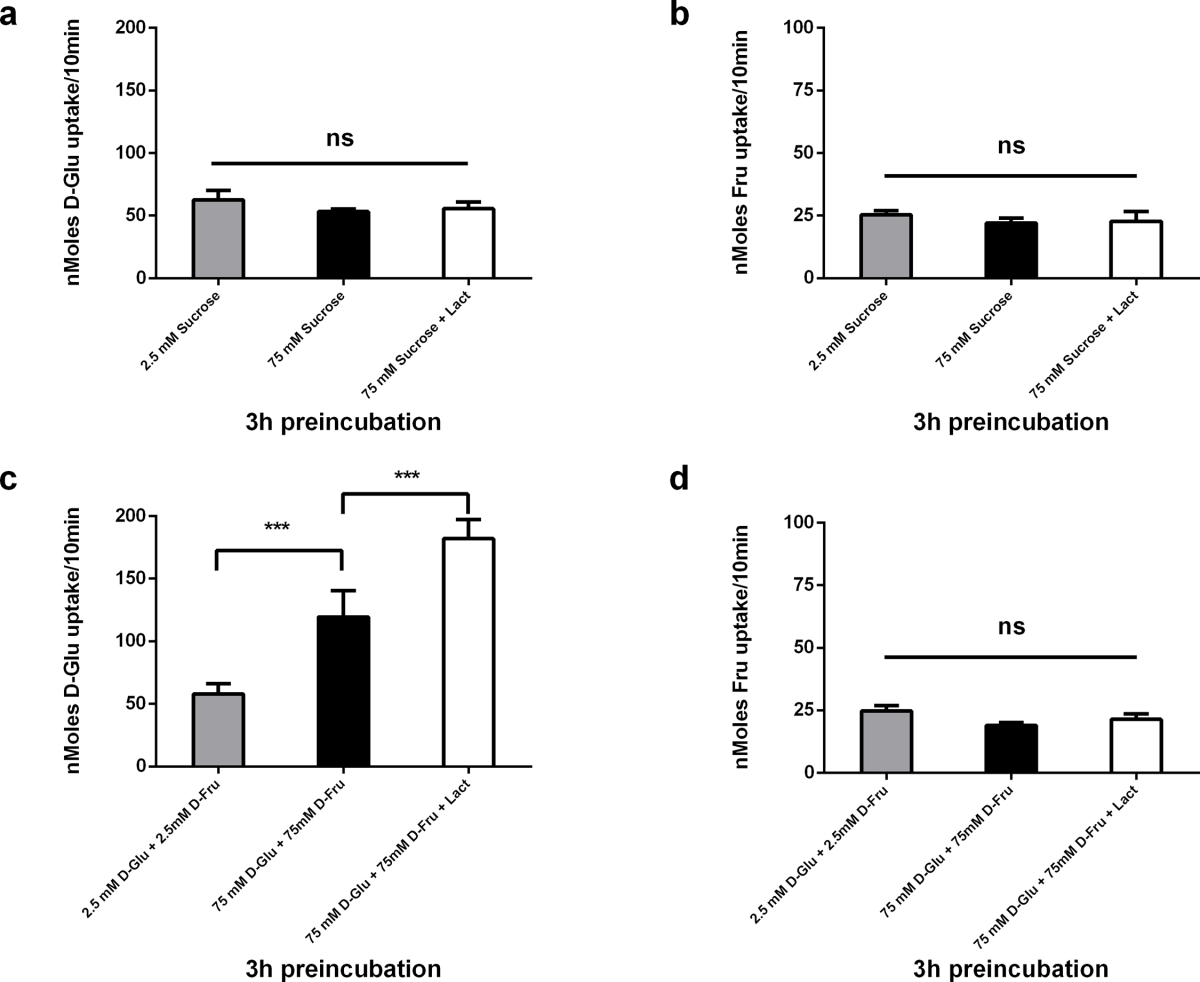
Acute effects of Sucrose, an equimolar mix of free glucose and fructose, and lactisole on carrier mediated [^14^C] 10 mM D-Glucose and D-Fructose uptake into Caco-2/TC7 cells. Caco-2/TC7 cells grown for 21 days were incubated with 2.5 and 75 mM Sucrose or a 50/50 mix of free D- Glucose/D-Fructose in the presence or absence of 0.5 mM lactisole for 3hrs, washed in sugar free KBS. (a) and (c) are cellular uptake of [^14^C] 10 mM D-Glucose for 10 mins (b) and (d) are cellular uptake of [^14^C] 10 mM D-Fructose measured by radioactive scintillation spectrometry. Osmolarity was adjusted by adding Mannitol. Uptake is corrected for simple diffusion of 10 mM [^14^C] L-Glucose. Data are expressed as nMoles sugar uptake/well/10min ± SD of n = 4 per condition. *P < 0.05, **P < 0.01, ***P < 0.001, ns = not significant.
