# Supplementary figures and images for: Acute Effects of Sugars and Artificial Sweeteners on Small Intestinal Sugar Transport: A Study Using CaCo-2 Cells As an In Vitro Model of the Human Enterocyte
Source: PLoS One. 2016 Dec 16;11(12):e0167785. doi: 10.1371/journal.pone.0167785 (PMC5161324; doi:10.1371/journal.pone.0167785)

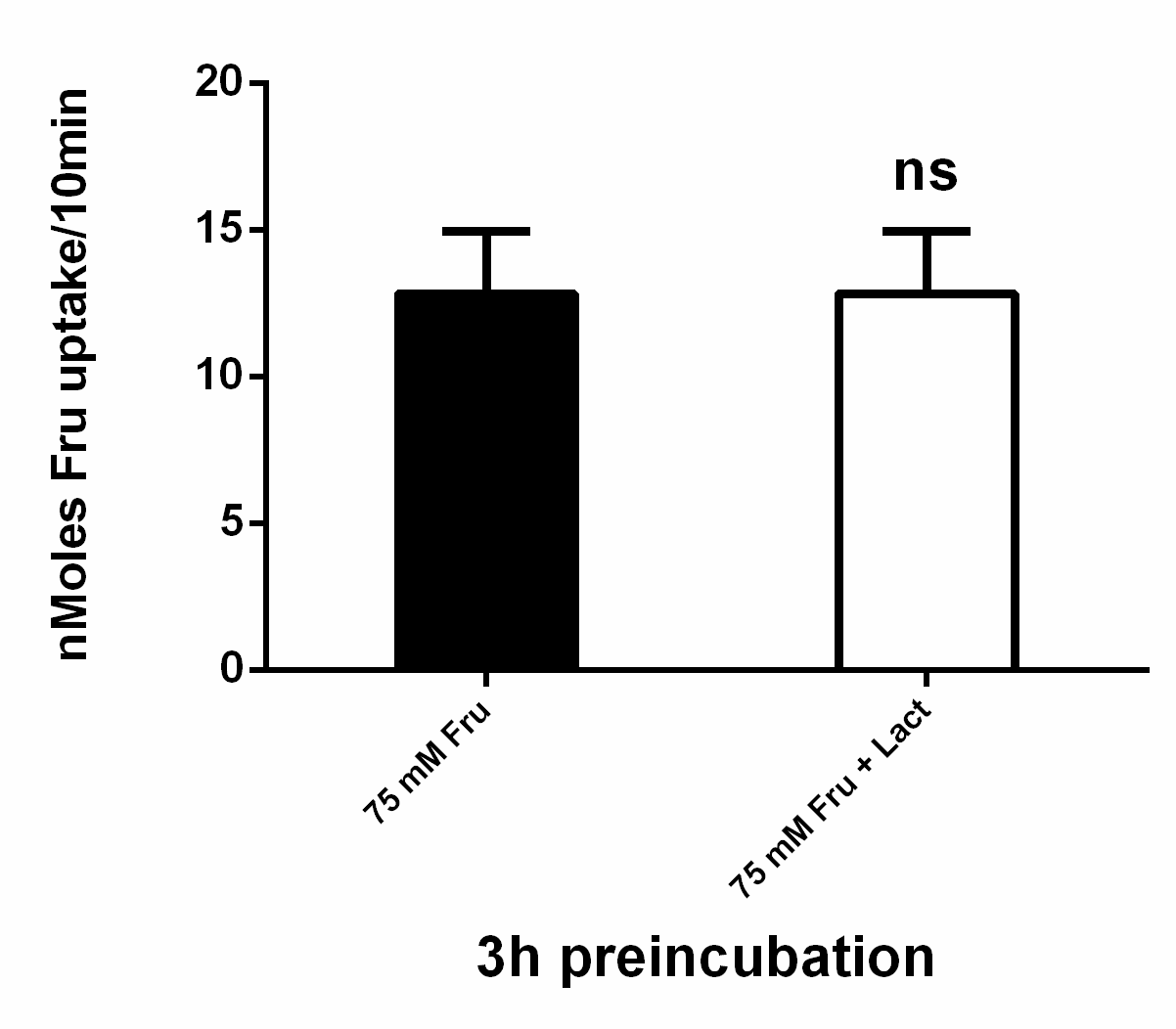

Supplement: S1 Fig — (a) Caco-2/TC7 cells grown for 21 days were incubated with 75 mM D-Fructose for 3hrs in the presence or absence of 0.5 mM lactisole, washed three times with glucose free KBS, followed by 10 min exposure to [14 C] 10 mM D-Fructose. Osmolarity was adjusted by adding Mannitol. Cellular uptake of D-Fructose was measured by radioactive scintillation spectrometry. Uptake is corrected for simple diffusion of 10 mM [14C] L-Glucose. Data are expressed as nMoles D-Fructose uptake/well/10min ± SD of n = 4 per condition. ns = not significant. (TIF) [file pone.0167785.s001.tif]

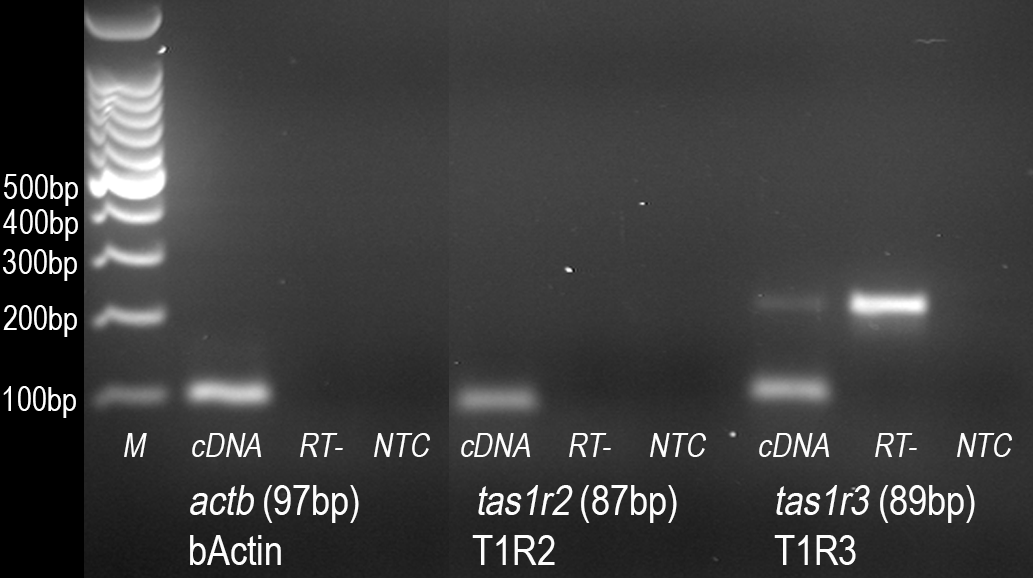

Supplement: S2 Fig — The presence of ß-actin, T1R2 (tas1r2) and T1R3 (tas1r3) transcripts in human tongue cDNA (Clontech) was assessed by PCR. (M) represents the molecular weight marker ladder. For each gene, (cDNA) represents a PCR amplification using tongue cDNA as a template, (RT-) represent a PCR using template obtained from a reverse transcriptase reaction without enzyme. (NTC) represents a PCR using no template. (TIF) [file pone.0167785.s002.tif]
